# Intranasal CEPO-FC prevents attention deficits in streptozotocin-induced rat model of Alzheimer's disease: Focus on synaptic plasticity-related factors

**DOI:** 10.17179/excli2023-6818

**Published:** 2024-04-22

**Authors:** Zahra Mansouri, Fariba Khodagholi, Jalal Zaringhalam, Fatemeh Abbaszadeh, Rasoul Ghasemi, Nader Maghsoudi

**Affiliations:** 1 Neuroscience Research Center, Shahid Beheshti University of Medical Sciences, Tehran, Iran; 2 Neurophysiology Research Center, Shahid Beheshti University of Medical Sciences, Tehran, Iran; 3 Department of Physiology, Faculty of Medicine, Shahid Beheshti University of Medical Sciences, Tehran, Iran; 4 Neurobiology Research Center, Shahid Beheshti University of Medical Sciences, Tehran, Iran

**Keywords:** Alzheimer's disease, attention, intranasal, CEPO-FC, STIM proteins, synaptic plasticity

## Abstract

Alzheimer's disease remains an issue of great controversy due to its pathology. It is characterized by cognitive impairments and neuropsychiatric symptoms. The FDA approved medications for this disease, can only mitigate the symptoms. One reason for the lack of effective medications is the inaccessibility of the brain which is encompassed by the blood-brain barrier, making intranasal (IN) route of administration potentially advantageous. Male Wistar rats underwent stereotaxic surgery to induce an Alzheimer's disease model via intracerebroventricular (ICV) streptozotocin injection, and Carbamylated Erythropoietin-Fc (CEPO-FC), a derivative of Erythropoietin without its harmful characteristics, was administered intranasally for ten consecutive days. Cognition performance for memory and attention was assessed using the Novel Object Recognition Test and the Object-Based Attention Test respectively. Depression like behavior was evaluated using the Forced Swim Test. Western blotting was done on the extracted hippocampus to quantify STIM proteins. Calbindin, PSD-95, Neuroplastin, Synaptophysin and GAP-43 genes were assessed by Realtime PCR. Behavioral tests demonstrated that IN CEPO-FC could halt cognition deficits and molecular investigations showed that, STIM proteins were decreased in Alzheimer's model, and increased after IN CEPO-FC treatment. Calbindin and PSD-95 were downregulated in our disease model and upregulated when treated with IN CEPO-FC. While Neuroplastin, and GAP-43 expressions remained unchanged. This study suggests that IN CEPO-FC in low doses could be promising for improving cognition and synaptic plasticity deficits in Alzheimer's disease and since IN route of administration is a convenient way, choosing IN CEPO-FC for clinical trial might worth consideration.

See also the graphical abstract(Fig. 1).

## Introduction

Alzheimer's disease (AD) affecting a growing number of adults, is predicted to rise to more than 150 million cases by 2050 (GBD 2019 Dementia Forecasting Collaborators, 2022[19]). Different types of cognitive alterations such as memory, concentration and attention deficits and neuropsychiatric symptoms (NPS) including depression, anxiety, aggression, agitation etc. are seen in almost all patients (Orgeta et al., 2017[46]) 

The causality of AD is under scrutiny and remains an issue of controversy. Consequently, there is no unanimity of a single mundane hypothesis. From a molecular point of view, Amyloid beta (Aβ) aggregation is known as the central pathological mechanism (Delport and Hewer, 2022[14]; Du et al., 2018[17]). There are also studies suggesting that the changes resulting from Ca^2+^ homeostasis disturbances, can precede Aβ aggregation and play a key role in AD pathophysiology (Chami, 2021[8]). 

Among the cognitive impairments in AD, memory deficit has been widely investigated but attention has not received as much attention despite being considered among the very first aspects affected by this disease (Malhotra, 2019[39]). Some believe that attention deficit might be the underlying factor of the inability of the sufferers to perform the memory tests, at least in the early stages (Bentley et al., 2008[6]).

In an endeavor to find different aspects of calcium signaling involved in neurodegeneration, various proteins have been scrutinized. Recently Store Operated Calcium Entry (SOCE) which is vital for synaptic plasticity (Zhang et al., 2016[70]) has gained considerable attention. This mechanism replenishes intracellular Ca^2+^, through its vital components which are stromal interaction molecules (STIM1 and STIM2). When endoplasmic reticulum (ER) Ca^2+^ depletes, STIM proteins are activated and they can move towards cell membrane and interact with ORAI channels (calcium release-activated calcium channel) leading to Ca^2+^ influx to compensate for the Ca^2+^ decrease (Popugaeva and Bezprozvanny, 2018[50]). Several studies have shown that, Ca^2+^ dysregulation mediated by STIM protein downregulation will result in cessation of calcium influx, necessary for the activity of Ca^2+^-calmodulin-dependent protein kinase II (CamkII) pathway (an essential process for memory formation). Therefore, the role of SOCE in general and STIM proteins in particular in neurodegeneration has been studied (Serwach and Gruszczynska-Biegala, 2020[58]) and assessing the beneficial or detrimental role of this protein, if changed, could be of great help to therapeutic perspectives.

To our knowledge STIM proteins have not previously been assessed in the streptoztocin (STZ)- induced rat model of AD nor have their alterations been assessed after Carbamylated Erythropoietin-Fc (CEPO-FC) treatment. In this study we analyzed the participation of STIM proteins alteration in inducing AD like symptoms and the probable role of CEPO-FC in modulating them. 

As it is stated by many studies, AD is a “lost memory” disease as a result of synaptic failure (Knobloch and Mansuy, 2008[29]). Synaptic plasticity involves communications between billions of neurons with essential post-synaptic density proteins located in the nanodomain of these connections that are crucial for synaptic stability and maintenance (Sheng and Kim, 2011[61]). PSD-95 is one of these proteins that dominates the complex, it connects to α-amino-3-hydroxy-5-methyl-4-isoxazolepropionic acid (AMPA) and N-methyl-d-aspartate (NMDA) receptors (Levy et al., 2022[35]) and controls the stability, engagement and transportation of proteins to post synaptic membrane. Therefore, it plays a vital role in synaptic plasticity and spine stability (Curran et al., 2021[13]). Studies have reported the decrease in PSD-95 in AD (Shao et al., 2011[60]).

Calbindin-D28k, maintaining calcium homeostasis in cells (Nanou and Catterall, 2018[45]), also has a role in synaptic plasticity and long-term potentiation (LTP) (Westerink et al., 2012[69]). As such this protein has been studied in AD (Kook et al., 2014[31]; Stefanits et al., 2014[64]). Other factors involved in synaptic plasticity include Synaptophysin, Neuroplastin and GAP-43. Synaptophysin, a presynaptic protein was shown to be reduced in AD (Masliah et al., 2001[40]). Neuroplastin is a cell adhesion glycoprotein belonging to the immunoglobulin superfamily, and is involved in synaptic formation and calcium signaling. It has a role in learning and memory (Ilic et al., 2021[26]) and has been linked to Alzheimer's condition, if there is any alteration in its expression (Ilic et al., 2019[25]). The growth-associated protein 43 (GAP-43), which is a presynaptic protein, is essential for learning and memory and regeneration (Qiang et al., 2022[52]; Sandelius et al., 2019[57]). With the advent of leisure in neurons, specifically in hippocampus new connections are built by GAP-43 involvement (Zhao et al., 2021[71]). The expression level of this protein has been shown to be altered paradoxically in AD (Dhiman et al., 2020[15]; Musunuri et al., 2014[43]). Since only a few FDA-approved drugs have been developed for symptom relief (Brunton et al., 2018[7]), various substances have been investigated, including erythropoietin (EPO). Although EPO can be lethal in effective doses, its derivatives have been successful in protecting the brain from neurodegeneration in many studies (Leist et al., 2004[34]). One of these derivatives is CEPO-FC which is produced by Lysin 7 carbamylization and FC attachment. This substance with a longer half-life and neuroprotective properties does not have hematopoietic effects (Leist et al., 2004[34]). Peripheral administration of this substance might be deleterious as it requires higher doses to penetrate the blood-brain barrier (BBB), therefore the intranasal (IN) route of administration is selected in this study. This method has many advantages, such as by passing the BBB, no enzymatic degradation, no hepatic first pass effect, rapid absorption and consequently rapid onset of function to name a few. Additionally, neurodegenerative diseases are usually progressive and thus need constant treatment hence the route of administration is of great importance so that the patients or caregivers can use the substance at home (Agrawal et al., 2018[2]; Alexander and Saraf, 2018[3]).

Given that no study is done on the effect of IN CEPO-FC on attention, memory loss and NPS seen in AD, therefore this study is to investigate whether intranasally administered CEPO-FC is capable of inhibiting these AD-like symptoms in a rat model by Novel Object Recognition Test (NOR), Object-Based Attention Test (OBAT) and Forced Swim Test (FST). Also, since synaptic plasticity failure as focused in this study, is a hallmark of cognition deficit and consequently AD, some synaptic plasticity-related proteins and genes that have been studied in AD as well, were selected to be assessed and find out if they can be affected by CEPO-FC. The factors are as follows; STIM1, STIM2, *Calbindin*, *PSD-95*, *Neuroplastin*, *GAP-43* and *Synaptophysin*.

## Materials and Methods

### Animals

Male Wistar rats (250-300 g), kept in groups of 3-4 in Plexiglas cages, were acquired from Pasteur Institute of Iran (Tehran, Iran). They were kept at an appropriate temperature (23±2 °C) and subjected to a 12-12-hour light-dark cycle, food and water were accessible ad libitum. 

### Materials

STZ was obtained from Sigma Aldrich Co., while CEPO-FC was made ready in our partner lab Vienna-Austria, Western blot antibodies β-Actin, STIM1 and STIM2 were acquired from Cell Signaling Technology Company. The Super RNA Extraction kit and cDNA Synthesis kit were purchased from Anacell Tehran, Iran. The 2x qPCR Master Mix Green-High Rox was obtained from Pishgam, Tehran, Iran, while the Primers were synthesized by SinaClon, Tehran, Iran. 

Animals were studied in 5 groups:

- Intracerebroventricular (ICV) Saline + IN Phosphate-buffered saline (PBS) as a vehicle for CEPO-FC (CONTROL)
- ICV STZ-induced AD model (STZ)
- AD model + IN CEPO-FC (STZ+CEPO-FC)
- AD model + IN PBS (STZ+VEHICLE)
- ICV Saline + IN CEPO-FC (CEPO).

### Drug preparation and administration

On the day of surgery, STZ was dissolved in saline at a concentration of 3 mg/kg (Duggal and Mehan, 2019[18]). CEPO-FC molecules were dissolved in PBS as the main stock at a concentration of 1.91 mg/ml (2.3x 10^5^ IU) and later it was diluted to concentrations of 25, 112, and 400 IU/kg for each administration which started on day 3 and continued consecutively for 10 days.

### Surgery

Rats were anesthetized using Ketamine (100 mg/kg) and Xylazine (10 mg/kg) intraperitoneal (IP) injection and fixed in a stereotaxic frame. Two holes were bored towards lateral ventricles (AP: -0.8; ML: ± 1.5; DV -3.5) (Javadpour et al., 2021[27]) based on Paxinos brain atlas. STZ was injected into ventricles using a Hamilton syringe at the speed of 1 µl/min to administer a precise dose. As of day 3, ketamine (25 mg/kg) was utilized to lightly anesthetize rats and CEPO-FC (25, 112, 400 IU/kg) was intranasally administered for 10 consecutive days, the protocol has been done previously in our collaborating laboratory (Pourhadi et al., 2023[51]).

For the CONTROL group normal saline was injected bilaterally into the ventricles and PBS was administered intranasally. For the CEPO-FC group, CEPO-FC was administered intranasally to rats, that had been intraventricularly injected with normal saline. 

### Behavioral tests

**Figure 2:**
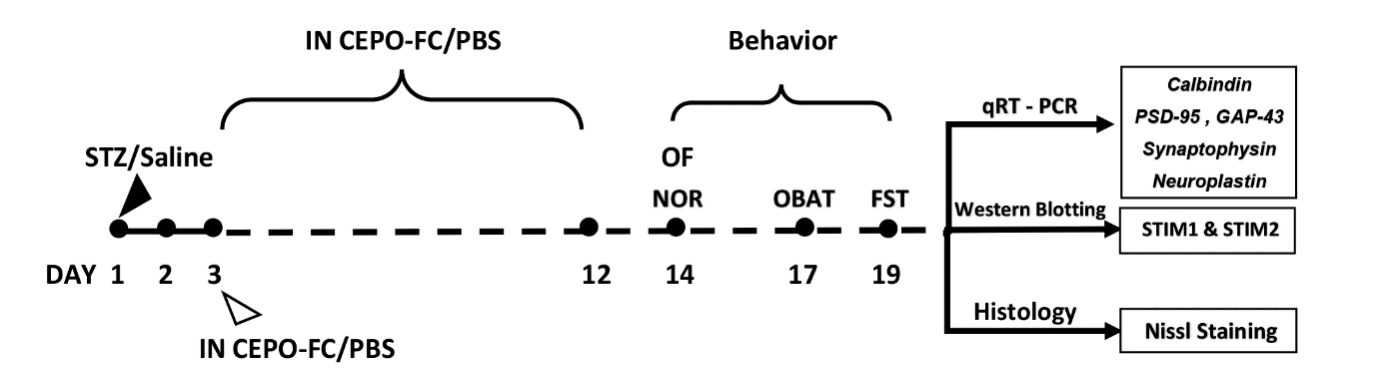
The study experimental diagram. STZ or saline was bilaterally injected intracerebroventricularly from day 3, and CEPO-FC or PBS was intranasally administered for 10 consecutive days. Behavioral tests were done 14 days after STZ injection. On the last day of behavioral tests, the animals were sacrificed and the hippocampus was extracted for further studies. STZ: Streptozotocin, IN: Intranasal, CEPO-FC: Carbamylated Erythropoietin-Fc , PBS: Phosphate-buffered saline, STIM: stromal interaction molecules, NOR: Novel Object Recognition test, OBAT: Object-Based Attention test, FST: Forced Swim Test, OF: Open Field test

Two weeks after surgery the tests were performed, the study planned procedure is shown in Figure 2(Fig. 2).

### Open Field Test

This test was done to evaluate any probable locomotor deficits caused by the procedure. Animals were put in a wooden box and videotaped for 5 minutes and then analyzed by Ethovision XT 11 (Noldus Information Technology, Wageningen, The Netherlands) software (Gould et al., 2009[21]).

### Novel Object Recognition Test

To evaluate animal's memory, NORT was performed based on the protocol provided by JOVE, with some modifications (Lueptow, 2017[38]). On day one, rats were placed in an empty wooden dark box for 5 minutes. After twenty-four hours, they were put in the same box with two similar objects for 5 minutes before being transferred to the cages. On day three the rats were returned to the box with one new object and one of the objects they were exposed to the day before. The behavior of rats during all stages of the test, was videotaped and analyzed. The total time spent investigating each object was considered, and object recognition index was determined as follows: time spent with the novel object on test day/ (time spent with the novel object+ time spent on familiar object on test day) *100.

### Object-Based Attention Test

OBAT was employed to assess animal's attention, which is simple and stress-free compared to the classic attention test (5-CSRTT). This test was conducted using the protocol presented by Alkam et al*.* (2011[4]) with some modifications. A rectangular PVC box divided into two parts (the training chamber, and the test chamber) was used for the test and the two parts were detached using a sliding door. On day one rats were put in the empty box. The sliding door was open and animals could freely navigate and move in both chambers for 10 minutes. Then the rats were taken back to their cages and after twenty-four hours they were put in the training chamber containing 5 objects that were different in shape but similar in size and color and material. After six minutes of exploration which was video-recorded, the objects were all collected and one of the them was transferred to the testing chamber (where a new object was already placed) parallel to its location in the training chamber and the sliding door was open so that the rat could go to the testing chamber. After entering, the sliding door was closed and the behavior of the animal was videotaped for 3 minutes. The cognition index for each rat was calculated: time spent with the novel object / (time spent with the novel objects+ time spent with familiar object) *100.

### Forced Swim Test

In order to assess depression like behavior in animals, FST was performed using a transparent Plexiglas cylinder containing fresh water at the temperature of 25^ °^C, on day one animals were placed in the cylinder (one by one) for 15 minutes. Twenty-four hours later the test was repeated and video-recorded for 5 minutes. The immobile time was measured. The animal was considered immobile when floating, almost upright and hands were not out of water or touching the walls to struggle (Slattery and Cryan, 2012[63]). 

After the test, anesthetized rats were decapitated and the hippocampi were extracted on ice for further experiments. The tissues were kept in liquid nitrogen for 24 hours and then in -80 °C.

### Western Blotting

In order to analyze the STIM1 and STIM2 proteins, the hippocampus was cold homogenized in a lysis buffer. After centrifuging for 30 min (13,000 rpm, 4 °C) the debris were removed. The protein was measured by Bradford assay. Loading sample buffer was added to the protein samples (40 µg each) and then the proteins in heated samples (100 °C for 5 min), separated by 12 % polyacrylamide gel electrophoresis, were transferred to PVDF membranes, which were then incubated for 90 min in a shaking incubator with 2 % skim milk.

Membranes were then probed overnight at 4 °C (STIM 1, STIM 2 and β-actin). The membranes were washed with TBST and then incubated for 1:15 hr. with horseradish peroxidase-conjugated anti-rabbit antibody. After incubation with ECL select kit (to visualize the immunoreactivity) the blots were ready. Finally, the radiographic films were used to calculate the quantification of protein band density by Image-J software (Hooshmandi et al., 2018[24]).

### Realtime PCR

The isolated RNA was quantified using a NanoDrop 2000 spectrophotometer (Thermo Scientific) and then used to synthesize cDNAs using the "cDNA synthesis kit" according to the instructions provided.

RT-PCR was utilized to evaluate the *Calbindin, PSD-95*, *Neuroplastin, Synaptophysin, GAP-43* and *β-Actin* expression levels. Briefly, cDNA products, specific primers, 2x qPCR Master Mix Green-High Rox and DEPC treated water, at a total amount of 10 µl were used to perform real-time PCR (Applied Biosystem StepOne Real-Time PCR System). After normalization with β-Actin as a control, the data analysis and mRNA level evaluation were performed using 2^−ΔΔCt^ values.

The primers of real-time PCR were as follows in Table 1(Tab. 1).

### Nissl staining

To perform morphological assessment, Nissl staining was used. Perfusion was done and the extracted whole brain was kept in 4 % paraformaldehyde for 24 hr, the brain underwent a dehydration process in graded alcohol solutions: 50 %, 70 %, 90 % alcohol and finally absolute alcohol.

Blocks were then prepared with pure melted wax. The whole brain was sectioned at seven micrometer thickness using rotary microtome. Following deparaffinization by xylene and ethanol, the sections were stained by 0.1 % Cresyl violet and then rinsed with distilled water and dehydrated in serial dilution of ethanol. Finally, they were submerged in xylene and, then cover-slipped (Miller et al., 2013[41]). The neuronal damages were evaluated in different areas of the hippocampus.

### Statistical analysis

Graph Pad Prism 8.0.2 was used to perform statistical analyses. Data are all shown as mean ± standard error (SEM) values. Normality tests of samples were passed by the Shapiro test. One-way ANOVA and Tukey's post hoc were used to show statistical differences between groups. P˂0.05 was considered as statistically significant.

## Results

**Figure 3:**
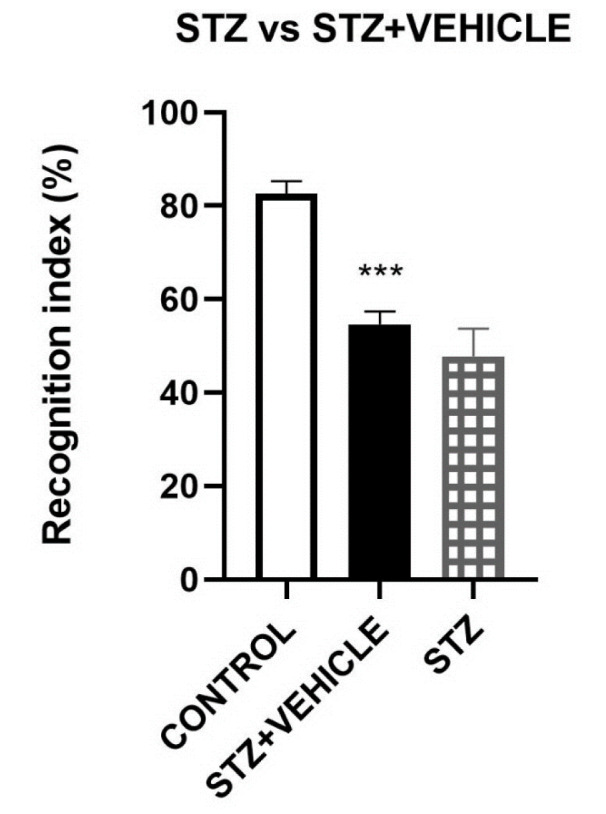
Comparison of STZ and STZ+VEHICLE. ^***^P < 0.001 STZ+VEHICLE vs CONTROL. Data are shown as Mean ± SEM (n = 8/group). STZ: Streptozotocin

Since the data gained for STZ and STZ+VEHICLE groups was not significantly different, only the STZ+VEHICLE were included in the rest of behavioral tests and molecular assessments (Figure 3(Fig. 3)).

### Intranasally administered CEPO-FC prevents Neuron damage as assessed by Nissl staining

**Figure 4:**
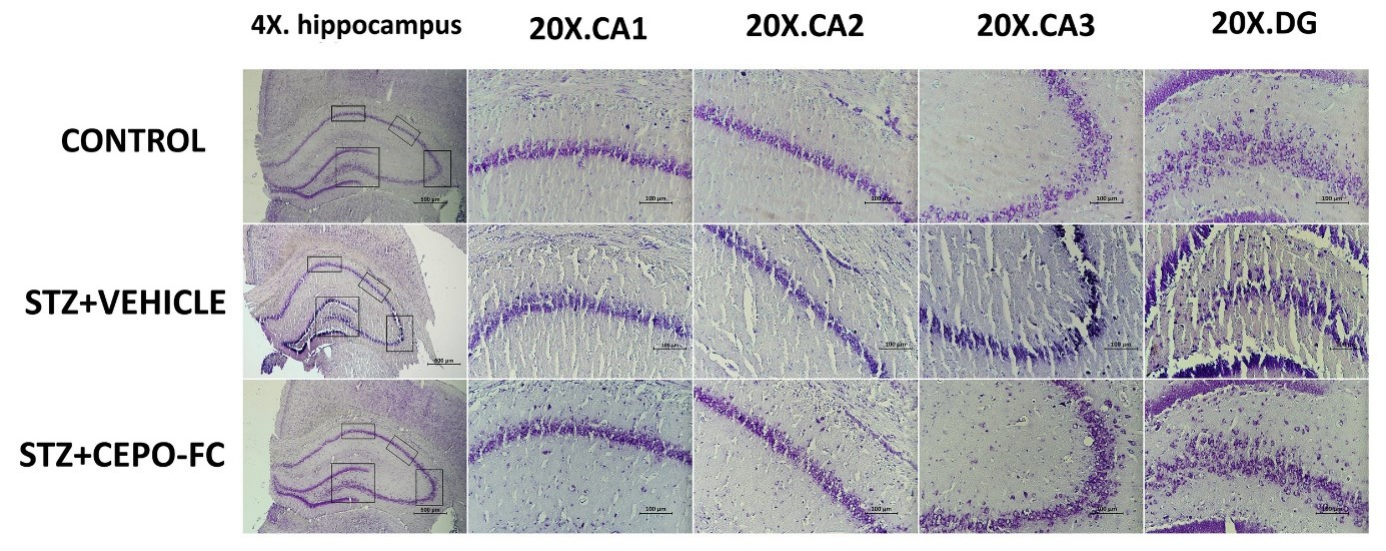
Neuronal injury shown by Nissl staining technique is represented in different groups at 4 hippocampus regions (CA1, CA2, CA3 and DG; at 2 magnificence (4x. and 20x.). The scale bars are 100 µm for the 20X figures and 500 µm for the 4X figures. STZ: Streptozotocin, CEPO-FC: Carbamylated Erythropoietin-Fc, DG: Dentate gyrus

Nissl staining technique was used to see neuronal damage in the hippocampus and as it is shown in Figure 4(Fig. 4), the neuronal damage is less in the hippocampus of the STZ +CEPO-FC and CONTROL group comparing to the STZ+VEHICLE group.

### Intranasally administered CEPO-FC halts STZ-induced memory impairments

**Figure 5:**
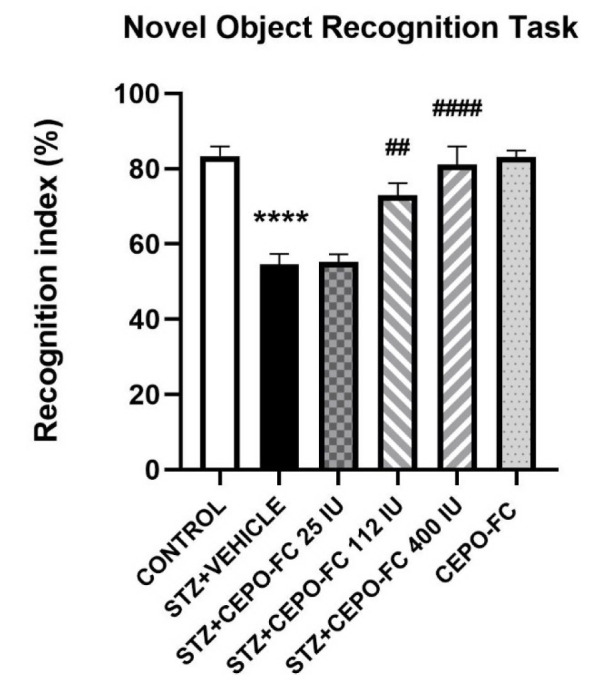
The effect of IN CEPO-Fc administration on STZ-induced AD models' NOR performance. The cognition index is lower in STZ treated group while IN CEPO-FC prevents the deficits. Data are represented as mean ± SEM. (*n* = 8/group).^ ****^P < 0.0001 STZ+VEHICLE vs CONTROL. ^##^*P* < 0.01 STZ+VEHICLE vs STZ+CEPO-FC 112 IU. ^####^*P* < 0.0001 STZ+VEHICLE vs STZ+CEPO-FC 400 IU. STZ: Streptozotocin, IN: Intranasal, CEPO-FC: Carbamylated Erythropoietin-Fc

The effect of intranasally administered CEPO-FC on STZ-induced memory impairments was investigated using the NORT 14 days after STZ or vehicle administration. As seen in Figure 5(Fig. 5), the cognition index in the STZ+VEHICLE group was lower than that of CONTROL (*P* < 0.05). The recognition index in STZ+CEPO-FC was higher than that of the STZ+VEHICLE group (*P* < 0.05) which is as a result of a 10-day IN treatment with CEPO-FC.

### Intranasally administered CEPO-FC halts STZ-induced attention impairments

**Figure 6:**
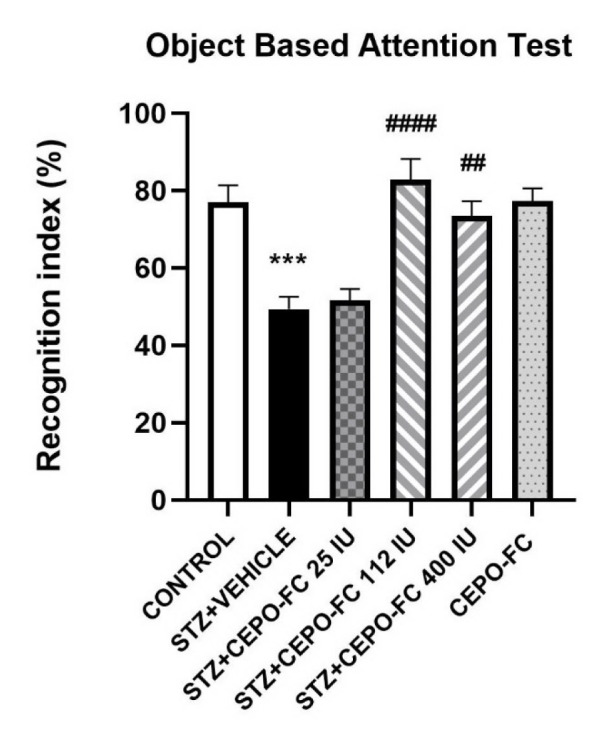
The effect of IN CEPO-Fc administration on STZ-induced AD models' OBAT performance. The cognition index is lower in STZ treated group while the deficits are halted by IN administration of CEPO-FC. Data are represented as mean ± SEM (*n* = 8/group).^ ***^P < 0.001 STZ+VEHICLE vs CONTROL.^ ####^*P* < 0.0001 STZ+VEHICLE vs. STZ+CEPO-FC 112 IU. ^##^*P* < 0.01 STZ+VEHICLE vs STZ+CEPO-FC 400 IU. STZ: Streptozotocin, IN: Intranasal, CEPO-FC: Carbamylated Erythropoietin-Fc

The effect of intranasally administered CEPO-FC on STZ-induced attention impairments was evaluated using OBAT. The cognition index which refers to animal's attention, significantly differed between groups as analyzed by one-way ANOVA. The cognition index in the STZ+VEHICLE group was lower than the cognition index seen in the CONTROL group (*P* < 0.05). Furthermore, rats with STZ-induced AD that were treated with CEPO-FC for 10 consecutive days showed a significantly better cognition index compared to the STZ+VEHICLE group (*P* < 0.05). This is demonstrated in Figure 6(Fig. 6).

### STZ and/or Intranasally administered CEPO-FC has no adverse effects on rats' locomotion

**Figure 7:**
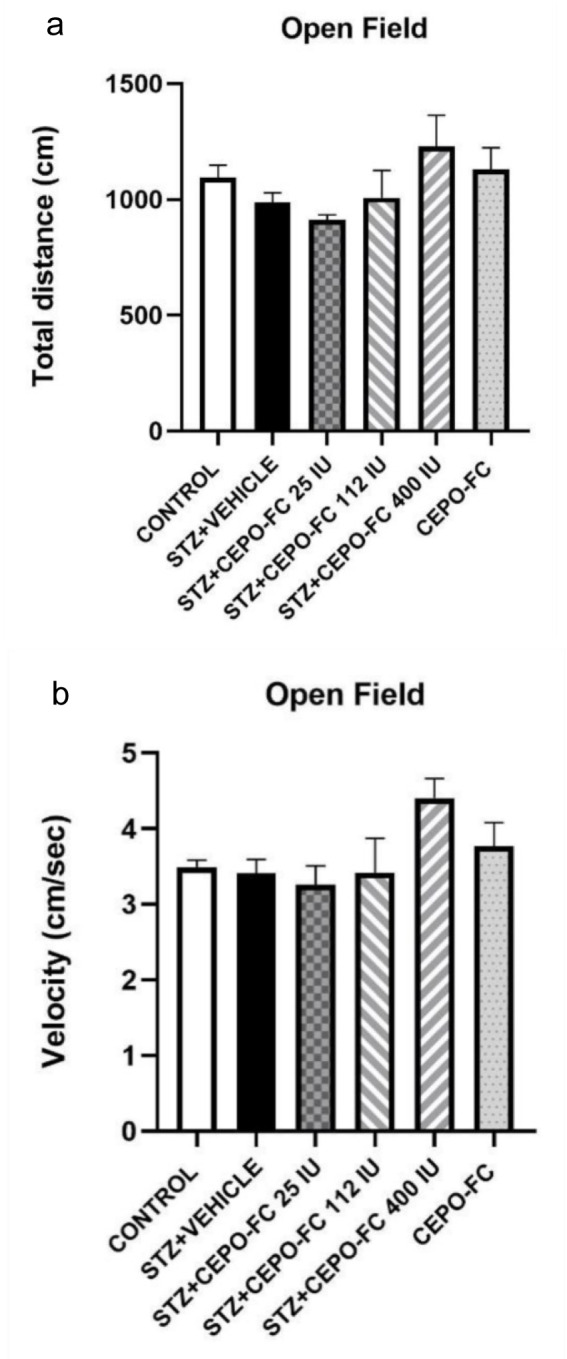
STZ and/or CEPO-FC administration has no adverse effect on animals' locomotion. a: Total distance. b: Velocity Data are presented as Mean ± SEM (*n* = 8/group). STZ: Streptozotocin, CEPO-FC: Carbamylated Erythropoietin-Fc

In order to evaluate the locomotor abilities of animals open field test was done. One-way ANOVA test showed no adverse effect of treatment with STZ and/or CEPO-FC on animal's locomotion (Figure 7(Fig. 7)).

### ICV administration of STZ-induces depression like behavior

**Figure 8:**
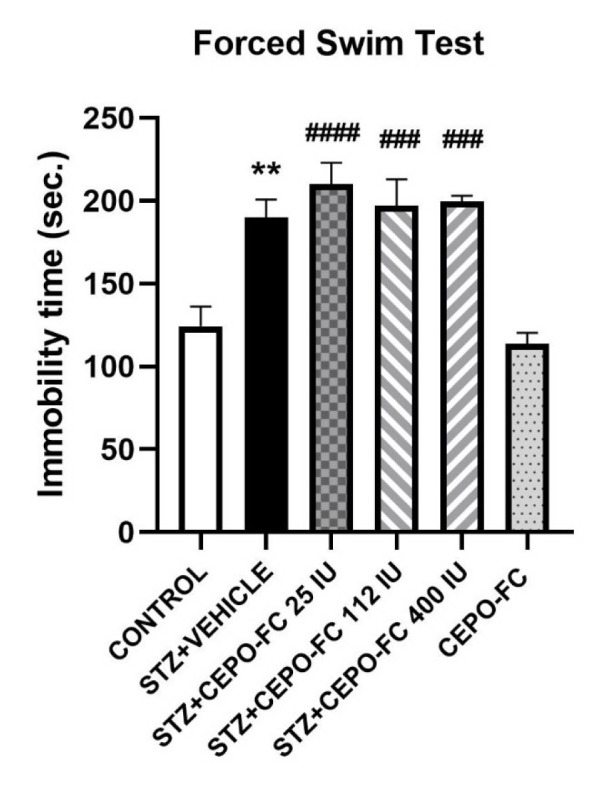
Effect of IN CEPO-Fc administration on depression like behavior of STZ-induced AD model. The immobility time is higher in STZ treated group. Data are shown as Mean ± SEM (*n* = 8/group). ^**^P < 0.01 STZ+VEHICLE vs CONTROL, ^###^*P* < 0.001 STZ+CEPO-FC 400 IU and STZ+CEPO-FC 112 IU vs CONTROL, ^####^*P* < 0.0001 STZ+CEPO-FC 25 IU. vs CONTROL. STZ: Streptozotocin, IN: Intranasal, CEPO-FC: Carbamylated Erythropoietin-Fc

The effect of ICV administration of STZ on depression-like behavior was evaluated using the Forced Swim Test, where the immobility time served as an index of depression-like behavior. One-way ANOVA demonstrated that the immobility time was more in the STZ+VEHICLE group in comparison to the CONTROL group. This is shown in Figure 8(Fig. 8).

### STIM1 and STIM2 proteins' expression changes were seen in STZ+VEHICLE and STZ+CEPO-FC groups

**Figure 9:**
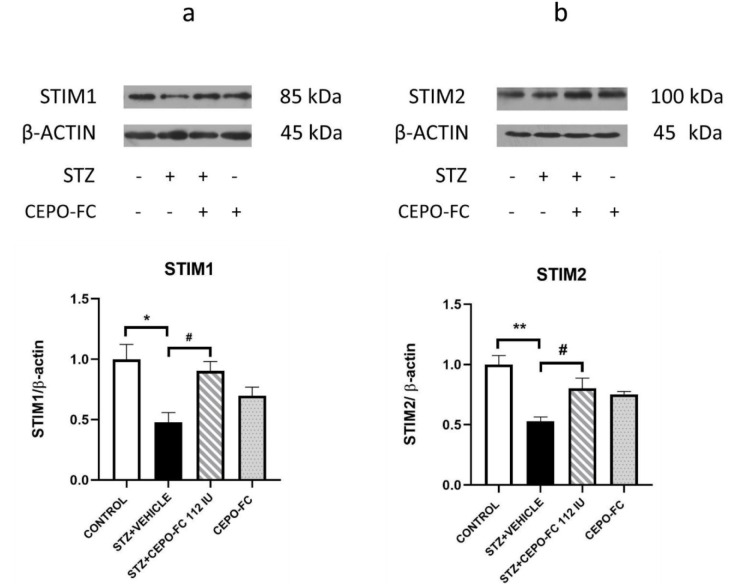
Alteration of Protein levels of STIM1 (a) and STIM2 (b) in the hippocampus following IN CEPO-FC administration. Data are represented as Mean ± SEM (*n* = 3/group). ^*^P < 0.05, ^**^P < 0.01 STZ+VEHICLE vs CONTROL, ^#^*P* < 0.05 STZ+VEHICLE vs STZ+CEPO-FC 112 IU. STZ: Streptozotocin, IN: Intranasal, CEPO-FC: Carbamylated Erythropoietin-Fc

As mentioned, STIM1 and STIM2 proteins are closely associated with synaptic plasticity. Therefore, in this study they were measured in the hippocampus using western blot analysis. One-way ANOVA demonstrated that levels of STIM1 were decreased in STZ-treated rats comparing to the CONTROL group (*P* < 0.05). Treatment with CEPO-FC, however, resulted in an augmentation of levels of STIM1 in comparison to the STZ+VEHICLE group (*P* < 0.05). The expression level of STIM2 in the hippocampus of the animals was also measured and found to be reduced in the STZ+VEHICLE group in comparison with the CONTROL group. Our data shows that the levels of STIM2 after treatment with CEPO-FC were also increased (*P* < 0.05, Figure 9(Fig. 9)).

### Alteration of PSD-95, Calbindin, and Synaptophysin expression shown by Realtime PCR

**Figure 10:**
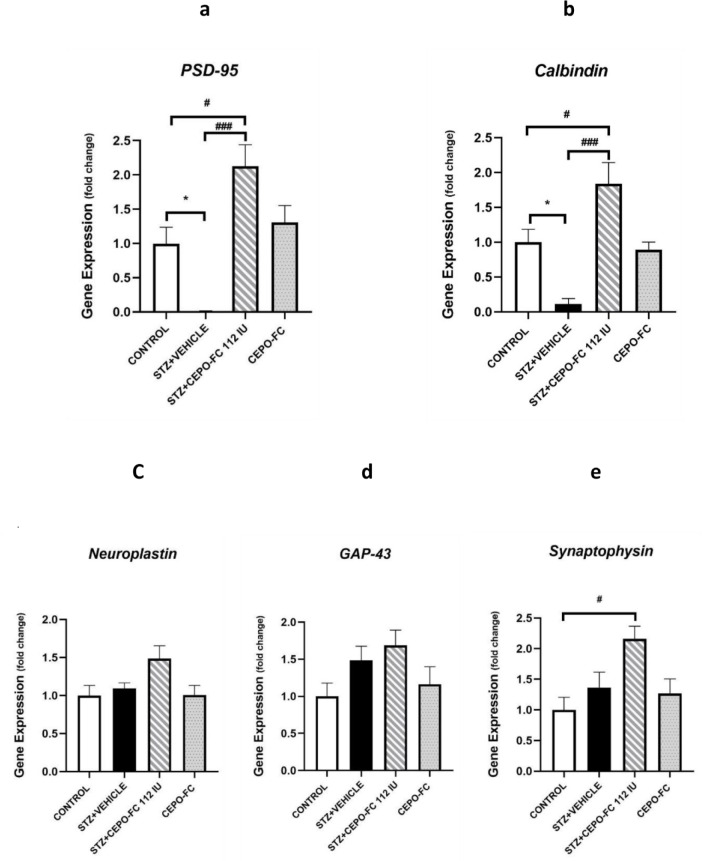
Changes in levels of synaptic plasticity-related genes expression (a: *PSD-95,* b: *Calbindin, *c: *Neuroplastin*, d: *GAP-43*, e:* Synaptophysin)* in the hippocampus following IN CEPO-FC administration. Data are presented as Mean ± SEM (n = 4/group). ^*^P < 0.05 STZ+VEHICLE vs CONTROL. ^###^*P* < 0.001 STZ+CEPO-FC 112 IU vs STZ+VEHICLE, ^#^*P* < 0.05 STZ+CEPO-FC 112 IU vs CONTROL. STZ: Streptozotocin, IN: Intranasal, CEPO-FC: Carbamylated Erythropoietin-Fc

The expression levels of five synaptic plasticity-related elements (*PSD-95,* *Calbindin,* *Neuroplastin,* *GAP43*, and *Synaptophysin*) were investigated using Realtime PCR. As shown in Figure 10(Fig. 10), the expression levels of *PSD-95* and *Calbindin* (*p* < 0.05) were lower in STZ+VEHICLE group in comparison with the CONTROL group. Additionally, the expression levels of *PSD-95* and *Calbindin* were significantly higher in the STZ+CEPO-FC group comparing to the STZ+VEHICLE group (*P* < 0.001) and the CONTROL group (*P *< 0.05). The expression level of *Synaptophysin* in the STZ+CEPO-FC group was also higher than that in the CONTROL group (*P* = 0.05). However, One-way ANOVA analysis showed no significant differences in the expression levels of *GAP43*, and *Neuroplastin* between any of the groups. 

## Discussion

This study investigated the potential role of intranasally administered CEPO-FC, in cognition deficit prevention in the AD model. The results show that IN CEPO-FC was able to halt the STZ-induced cognition impairments. The dose of CEPO-FC used in this work was obtained in a pilot study conducted previously in our laboratory. We also examined the doses 25 IU and 400 IU, but no significant changes were observed at 25 IU, and no significant difference from the 112 IU dose was seen at 400 IU. Therefore, only the 112 IU dose was used for molecular and histological assessments.

The attention deficit induced by ICV injection of STZ could be halted by IN CEPO-FC administration. This result is consistent with some former studies. A study suggested that the AD animal models exhibit both memory and attention deficits (Romberg et al., 2011[53]). Malhotra suggested that AD is primarily a memory disease with obvious impairments in attention while some early symptoms attributed to episodic memory might be as a consequence of impairment in attention system in patients (Malhotra, 2019[39]). Since one of the most important pathways involved in ADHD is the disturbance in Ca2 activated potassium channel (Mooney et al., 2016[42]) and Ca^2+^ dysregulation is one of the main pathological hypotheses of AD, it can be inferred that attention impairment could be one of the important symptoms of AD. Additionally it has been shown that the most strongly associated locus with ADHD is in STIM1 (Grove et al., 2019[23]). Since STIM 1 protein was decreased in our STZ+VEHICLE group and upregulated in CEPO-FC treated group, it can be concluded that STIM1 can be a candidate protein to be investigated more as a molecule to be involved in attention. Additionally, since CEPO-FC was shown to modulate some synaptic plasticity genes (Tiwari et al., 2021[66]) and at the same time, STIM proteins which have been shown to be involved in AD (Chami and Checler, 2020[9]) are presented to be involved in synaptic plasticity (Korkotian et al., 2017[32]; Zhang et al., 2016[70]), STIM1 and STIM2 were quantified in our work.

To our knowledge this work is the first report of STIM proteins alteration in an STZ- induced rat model of AD. It was also shown that IN administered CEPO-FC possibly exerts its effects at least partially, through increasing STIM proteins. 

In a 2016 study, it was stated that mutant PS1 hippocampal neurons had deficits in Ca^2+^ signaling which were rescued when STIM1 was overexpressed (Tong et al., 2016[67]). Another study demonstrated that the expression of FAD-PS1 and -PS2 results in the reduction of SOCE through downregulation of STIM1 (Greotti et al., 2019[22]). In 2014, a group of researchers showed that SOCE mediated by STIM2 and consequently the activity of CaMKII is responsible for mushroom spines' stability. They demonstrated that STIM2 reduction causes a deficit in STIM2-nSOC-CaMKII pathway; this deficit is seen in sporadic AD brains (Sun et al., 2014[65]). In a recent study Naderi et al*.*, also demonstrated that STIM1 and STIM2 are significantly reduced in Aβ treated rats which could be due to ferroptosis or necroptosis (Naderi et al., 2023[44]). These results are in agreement with our findings. In 2018, 2019 and 2022, Skobeleva et al*. *(2022[62]; Chernyuk et al., 2019[10]; Ryazantseva et al., 2018[55]) working on familial models of AD claimed that STIM mediated SOCE is enhanced in AD model. This contradiction might be because they have assessed familiar Alzheimer's model with different gene mutations, while AD-like symptoms induced by STZ usually represent sporadic AD (Rostami et al., 2017[54]).

In this study it was shown, that CEPO-FC might prevent cognition impairment by upregulating Calbindin and PSD-95 genes which were downregulated in our AD model. This result is in accordance with other studies which investigated these molecules. It has been shown by Palop et al*.*, that in hAPP mice with elevated levels of hippocampal Aβ, Calbindin levels are decreased in dentate granule cells. They also found that the depletion is seen in the dentate gyrus of AD patients (Palop et al., 2003[47]). In addition, *Calbindin* mRNA levels in the hippocampus were assessed and found to be reduced in hAPP mice, which shows that calbindin reductions are resulted from lower expression levels of the *Calbindin* (Chin et al., 2008[11]). In a study conducted by Kook in 2014, they suggested that loss of Calbindin has a crucial role in AD pathogenesis (Kook et al., 2014[31]). In 2024 Acharya et al. have related low vitamin D and calbindin to AD pathology (Acharya et al., 2024[1]).

The other synaptic plasticity-related factor assessed in this study was *PSD-95*, the expression of which was decreased in the STZ+VEHICLE group and increased in CEPO-FC treated rats. A study reported that *PSD-95*^−/−^ mice show deficits in learning and working memory and in general lack of *PSD-95* may lead to disruption in synaptic functions (Coley and Gao, 2019[12]). The results obtained from the study conducted by Kim Dore, reveal that increased PSD-95 can prevent Aβ toxicity for synapses, this also suggests that reduction in levels of PSD-95 may be involved in AD and increases in PSD-95 levels at synapses prevent the synaptic deficits caused by Aβ (Dore et al., 2021[16]). Another study also shows PSD-95 decrease in the hippocampus of a mouse model of AD which they believe is induced by either Aβ or tau (Shao et al., 2011[60]); these results are in agreement with ours.

A couple of studies on the role of Neuroplastin and Synaptophysin in AD state that they are both reduced in AD patients (Ilic et al., 2019[25]; Poirel et al., 2018[49]). For GAP-43 there are conflicting reports that have shown either upregulation or downregulation or even stable levels of this protein based on the different areas of the brain assessed (Lee et al., 2023[33]; Musunuri et al., 2014[43]). While all these studies (on Neuroplastin, Synaptophysin and GAP-43) were done on postmortem human tissues, the inconsistency between the results of these studies and ours may be due to the differences in mechanisms underlying the induction of AD-like symptoms by STZ. Additionally, in the mentioned studies these factors were assessed at protein levels which may suggest that the protein decrease might be due to posttranslational deficits. Since GAP-43 is also a regeneration factor, in our study this protein seems to have an increasing trend in the STZ+VEHICLE group indicating that sprouting might be occurring, albeit not significantly. 

On the other hand, the expression levels of all investigated synaptic-related factors appeared to have an increasing trend in CEPO-FC treated rats regardless of whether they were changed in the STZ+VEHICLE group or not. In physiological conditions, double strand breaks (DSBs) occur and DNA repair mechanisms are activated. Furthermore, NMDA activity, which is an important factor in learning and memory, induces DSBs and consequently repair mechanisms are initiated. These events are essential for neuronal viability and are collectively referred to as the DNA Damage Response (DDR) (Konopka and Atkin, 2022[30]). In some recent studies it was shown that learning can also cause DSBs, therefore DNA damage could lead to facilitation of many functions in neurons. Many researchers are of the opinion that aging and AD may result from the accumulation of unrepaired DNA damages (Lin et al., 2020[36]; Shanbhag et al., 2019[59]; Welch and Tsai, 2022[68]). At the same time in aging and AD synaptic plasticity and DNA repair-related genes are downregulated (Konopka and Atkin, 2022[30]). In CEPO-FC treated rats the probable DNA damage produced by STZ leads to activation of DDR and the presence of CEPO-FC prevents the DNA repair mechanisms from failing and ultimately leading to upregulation of synaptic plasticity genes. In fact, in the CONTROL and CEPO-FC groups, there was no trigger to enhance DDR. The mechanism by which CEPO-FC might have promoted DDR is unknown as it was not investigated in this study but considering the fact that AKT has a role in DNA repair (the exact and detailed mechanism is still unknown) (Liu et al., 2014[37]) and its over-activation suppresses DNA repair (Piscitello et al., 2018[48]). We can refer to the previous work done in our laboratory (Hooshmandi et al., 2018[24]), where we found that treatment with CEPO-FC (in Aβ treated animals) decreased this protein kinase. Therefore, it may be hypothesized that CEPO-FC may promote DNA repair by somehow decreasing AKT.

A study in 2015 found that chronic treatments with IP injection of CEPO improved memory in the APP/PS1 mouse model of familial AD (Armand-Ugon et al., 2015[5]). Similarly, Hooshmandi and colleagues showed that IP CEPO-FC could prevent cognition impairments in Aβ-induced rats model of AD (Hooshmandi et al., 2018[24]). These results are consistent with our results. It is worth noting that all previous studies used IP CEPO-FC while our results show that IN administration of CEPO-FC has the same neuroprotective effects even at lower doses.

IN CEPO-FC could not stop the occurrence of depression in our AD model. A study in 2020 showed that IP CEPO-FC has anti-depressant effects (Sampath et al., 2020[56]). Our results are not in agreement. This discrepancy may be because the anti-depressant effect seen in the mentioned study was not on the AD model. As it is stated, the mechanism underlying depression in AD might be different, so the impairments in pathways involved in depression in AD, at least in our disease model, did not overlap with pathways covered by CEPO-FC. Also, our route of administration (IN) was different. 

**Figure 11:**
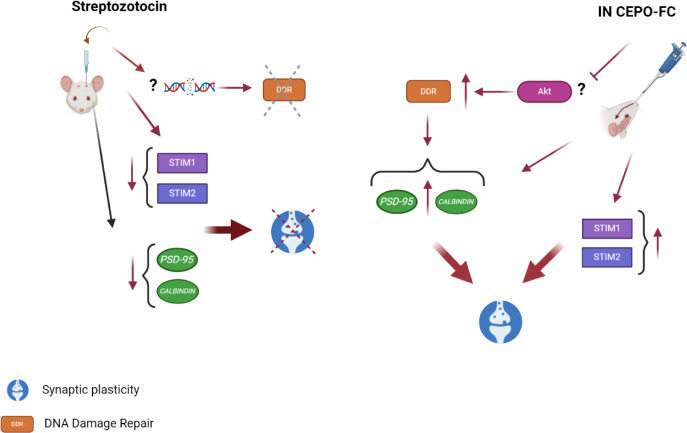
Suggested mechanistic summary of how CEPO-FC may have affected synaptic plasticity, which is through upregulation of STIM proteins and PSD-95 and Calbindin genes and probably by decreasing the AKT activity, CEPO-FC has enhanced the DNA damage repair which in turn upregulates synaptic plasticity factors (created with biorender.com).

Considering the results gained in this study, the authors suggest a mechanistic summary shown in Figure 11(Fig. 11).

## Conclusion

In this work CEPO-FC was shown to be an efficacious substance in forestalling AD deficits when administered intranasally in rats. As a matter of fact, CEPO-FC, prevented synaptic failure and modified STIM proteins and Calbindin, and PSD-95 gene expressions. In order to have a better understanding of the mechanism of action of CEPO-FC in the field of attention, it is recommended to assess the other specific pathways and molecules directly involved in attention. This is a promising result because one of the most important factors in the treatment or preventative measures for progressive conditions, is the ease of use for sufferers or caregivers. Overall, these results suggest that IN CEPO-FC has potential as a therapeutic substance in AD treatment and warrants further investigations. Now that many ways are being developed to predict AD years before the onset of the disease (Jonaitis et al., 2023[28]), substances such as CEPO-FC as neuroprotective agents (which can halt deficits), and easy to administer, are of great importance to be considered.

## Notes

Rasoul Ghasemi and Nader Maghsoudi (Neuroscience Research Center, Shahid Beheshti University of Medical Sciences, Chamran Highway, Velenjak, Tehran, Iran; E-mail: nmaghsoudi@sbmu.ac.ir) contributed equally as corresponding author.

## Declaration

### Ethics approval

The protocols were approved by the ethics committee of Shahid Beheshti University of Medical Sciences (IR.SBMU.PHNS.REC.1400.006) and all guidelines for care and use of laboratory animals were observed (National Institute of Health Publication, No.80-23, revised 1996).

### Acknowledgments

The authors wish to express their gratitude to Shahid Beheshti University of Medical Sciences for funding this study as part of the Ph.D. dissertation of Zahra Mansouri (Grant number: 25010) and supporting of the Neuroscience Research Center of Shahid Beheshti University of Medical Sciences. 

## Figures and Tables

**Table 1:**
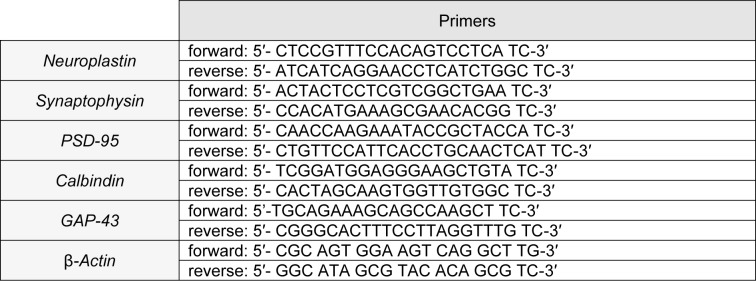
The table shows forward and reverse primers for each gene.

**Figure 1:**
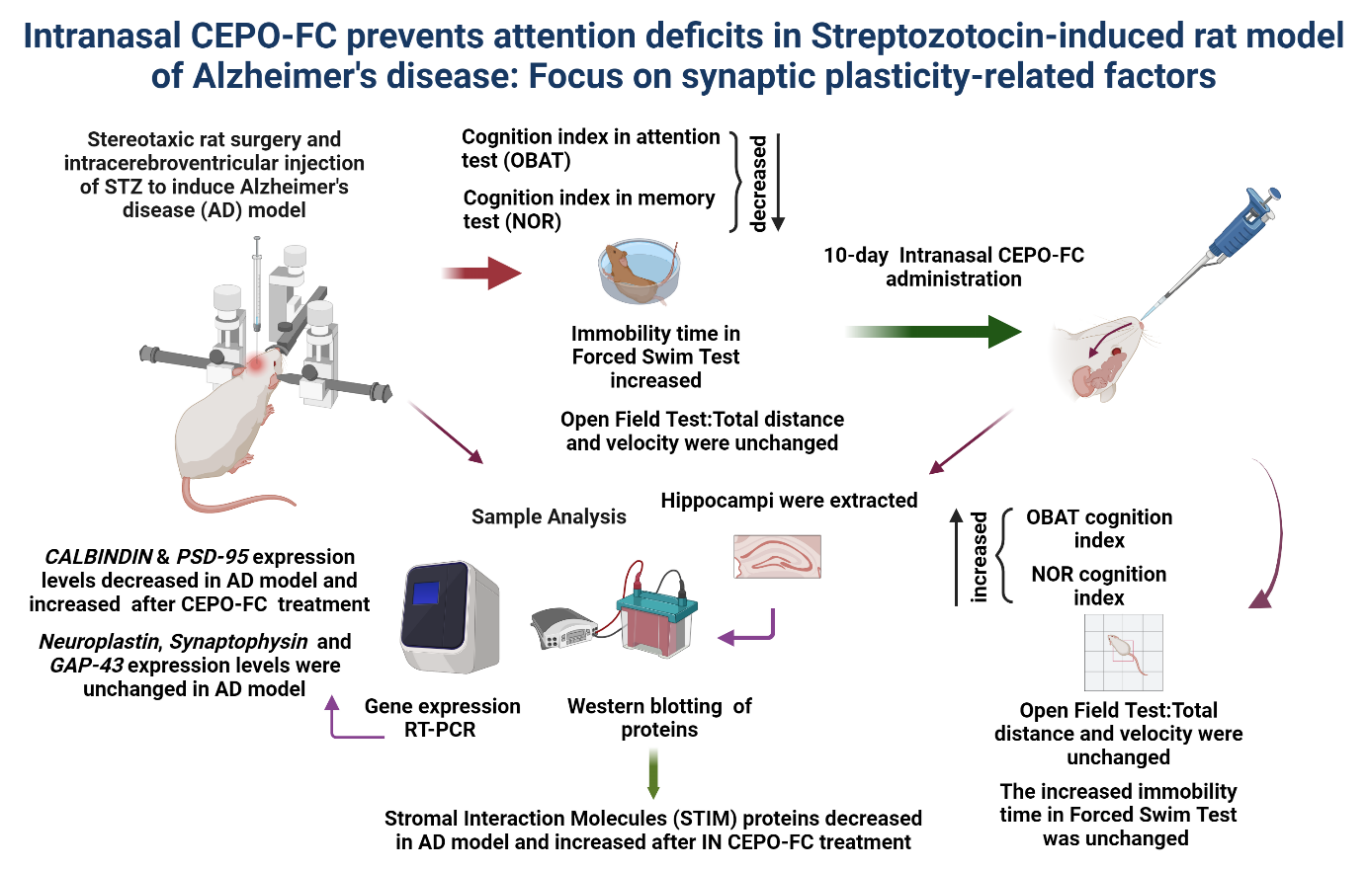
Graphical abstract (created with biorender.com)
